# A mathematical model for designing a reliable cellular hybrid manufacturing-remanufacturing system considering alternative and contingency process routings

**DOI:** 10.1007/s42452-021-04315-y

**Published:** 2021-02-22

**Authors:** Amirreza Hooshyar Telegraphi, Akif Asil Bulgak

**Affiliations:** Department of Mechanical, Industrial, and Aerospace Engineering, Gina Cody School of Engineering and Computer Science, Concordia University, Montreal, QC H3G 1M8 Canada

**Keywords:** Sustainable manufacturing, Cellular manufacturing systems, Alternative routing, Contingency routing, Reliability, Closed-loop supply chain

## Abstract

Due to the stringent awareness toward the preservation and resuscitation of natural resources and the potential economic benefits, designing sustainable manufacturing enterprises has become a critical issue in recent years. This presents different challenges in coordinating the activities inside the manufacturing systems with the entire closed-loop supply chain. In this paper, a mixed-integer mathematical model for designing a hybrid-manufacturing-remanufacturing system in a closed-loop supply chain is presented. Noteworthy, the operational planning of a cellular hybrid manufacturing-remanufacturing system is coordinated with the tactical planning of a closed-loop supply chain. To improve the flexibility and reliability in the cellular hybrid manufacturing-remanufacturing system, alternative process routings and contingency process routings are considered. The mathematical model in this paper, to the best of our knowledge, is the first integrated model in the design of hybrid cellular manufacturing systems which considers main and contingency process routings as well as reliability of the manufacturing system.

## Introduction

Sustainability is one of the significant criteria for companies to be successful in their promotion and selling activities. Along with the environmental regulations and the societal pressure from people in the form of community associations, the main motivation for companies to be sustainable is the potential economic advantages [1]. Sustainability as a multi-dimensional perception contains various meanings including green, clean, maintain, retain, stability, ecological balance, natural resources and environment [2]. In manufacturing systems, sustainability deals with the design of a production system emphasizing the three pillars of sustainability, namely economy, society, and environment [1]. Designing sustainable manufacturing needs a holistic insight over the manufacturing enterprise which one needs to consider all the components in the design of a supply chain including production facilities, customers, collection facilities, and disassembly facilities [3]. Sustainability is a prevalent research field in the design of closed-loop supply chain and reverse logistics. Designing sustainable manufacturing systems is increasingly absorbing attention in both research and academia. The cellular manufacturing system is an application of group technology in which similar parts are assigned into part families to take advantage of similarities in production and design and different machines are assigned into machine cells based on alike processing requirements of the parts [4]. The cellular manufacturing system in this paper considers reconfigurability issues in which the physical structure(s) of the system can be adjusted fast to improve the capacity and functionality managements. According to Garbie et al. [3], for designing a sustainable manufacturing system, dynamic cellular manufacturing can be applied to improve the pillars of sustainability due to mass customization. Accordingly, producing products and services for the individual needs of customers implies a significant reduction in the total wastes which results in the positive environmental effects as well as the increased customers’ satisfaction. Moreover, mass customization increases the efficiency of production [3]. According to Wang et al. [5], resorting to digital twin manufacturing with the aim of big data analytics, internet of things (IoT), edge computing and artificial intelligence through the use of self-thinking, self-decision making, self-execution and self-improving would be able to increase the quality and throughput of the manufacturing systems, while keeping adequate flexibility and reducing cost. Hence, resorting to digital twin manufacturing improve the economic and environmental pillars of the sustainable manufacturing systems. The implementation of blockchain technology as an enabler to drive existing manufacturing information systems such as enterprise resource planning (ERP) and manufacturing execution system (MES) also could also strengthen the sustainability pillars through energy-saving and energy-conserving benefits in designing manufacturing systems [6]. “The presence of alternative processes routings is typical in many discrete, multi-batch, small lots size production environments” [7]. Resorting to alternative process routings increases the number of ways to form the manufacturing cells [7]. Contingency process routing is a particularly important manufacturing attribute that has been extensively studied by Ahkioon et al. [4] to increase the flexibility and reliability in the design of cellular manufacturing systems. In designing sustainable manufacturing systems, hybrid manufacturing-remanufacturing systems can also be applied because of their social, economic and environmental impacts. Remanufacturing is an industrial process in which worn-out products are restored to like-new conditions [8]. Remanufacturing is one of the key issues for companies to bring the returned products with high quality to the functional state in which the reliability of the remanufactured products is sometimes superior to the original new product [9, 10]. Remanufacturing cost of returned products is on average 40–60% less than manufacturing cost of the new products [11]. Accordingly, remanufactured products are sold, with the same warranty as for new products, with a price discount between 30 and 40%. There are also different types of customers, namely newness-conscious and functionally oriented customers [12]. Newness-conscious customers consider lower value for remanufactured products, and they even may not buy remanufactured products at all. However, functionally oriented customers understand that the quality of the remanufactured products is comparable or sometimes superior to the original new products, so they tend to buy remanufactured products [12]. Functionality oriented customers are going to buy new products if remanufactured products are not offered [12]. Therefore, there are different demand flows for new and remanufactured products. Remanufacturing activities can be done in remanufacturing facilities or hybrid manufacturing-remanufacturing facilities with shared resources. In recent years, designing hybrid manufacturing-remanufacturing systems has become a topic of substantial interest due to economic opportunities, social incentives, and environmental legislation. There are many companies in the fields of automobile parts, industrial engines, computers, cellphones, cameras, and copiers which adopted products recovery activities such as remanufacturing as a value-added strategy including D&V Electronics, JOHN DEERE Tractors and Dozers, Caterpillar, OEM Remanufacturing, Dell, HP, Dexter, Xerox, and Kodak. For example, Caterpillar Inc as a producer of machinery, engines, and financial products started remanufacturing activities in 1972. In 2007, the estimated profits of Caterpillar’s remanufacturing division were over $2 billion dollars as the fastest growing divisions in the company. As another example, Dexter, Michigan-based ReCellular sells about 4 million remanufactured cellphones worldwide each year [5].

The remainder of the paper is organized as follows: detailed description of the mathematical model is presented in Sect. 3. Solutions as well as a detailed economic analysis of the model on an example problem are presented in Sect. 4. A detailed sensitivity analysis is done in Sect. 5 including 7 different scenario problems to investigate the impacts of changing the number of time periods, number of the cells, number of the machines, and number of the returned products on the objective function value as well as the solution time. Also, the impacts of the consideration of alternative and contingency process routings on the objective function value are investigated in Sect. 5. In Sect. 6, conclusions and future research are presented.

## Literature review

In this section, research related to dynamic cellular manufacturing systems and hybrid manufacturing-remanufacturing is reviewed. Due to abundance of the related research in these topics, we only focus on the design optimization and mathematical modeling of these systems.

### Cellular manufacturing systems

Mathematical programming is used massively in designing of the cellular manufacturing systems. The objective function of the mathematical models is usually to minimize the total costs of the system related to the operational aspects of the system. Purchek [13] introduced, for the first time, the use of linear programming in the mathematical modeling of a cellular manufacturing system. The main objective of the linear programming (p-median) model of the Purcheck, considering the concept of power sets in Boolean Algebra, was clustering of parts to part families and machines to machine cells. Ben-Arieh and Sreenivasan [14] analyzed the effects of the information available including the relevant features of the parts for clustering the components in a dynamic cellular-line manufacturing system. They proposed a grouping methodology (i.e. negotiation-based dynamic clustering) that allows the parts to be grouped as they arrive in a piece-wise manner. Their algorithm has two stages in which the agents are assigned to each part type as well as each part type to a group in the first stage and opening the negotiations among the agents to optimally allocate parts to the groups in the second stage. Solimanpur et al. [15] addressed the material handling issues such as inter-cell and intra-cell material handling in the design of a dynamic cellular manufacturing system. They developed a two-stage heuristic, namely SVS-algorithm to efficiently solve their mathematical model. Chen and Cao [16] presented a mathematical model considering production planning, cell formation problem, and fixed costs at the same time considering quadratic terms for the material handling issues in the objective function toward designing a cellular manufacturing system. Their model encompasses minimizing a number of cost elements including material handling between cells, manufacturing setup, cell setup, inventory holding and production planning. They solved the mathematical model with the use of LINDO optimization software for small instances and a Tabu-search heuristic for the large-sized instances. Defersha and Chen [7] proposed a comprehensive mixed-integer mathematical model for designing a dynamic cellular manufacturing system. Their model encompasses decisions related to the dynamic cell configuration, alternative routings, lot splitting, sequence of operations, multiple copies of identical machines, machine capacity, workload balancing among the cells, operational costs, subcontracting cost of the part demands, production cost per unit, tool consumption cost, setup cost, cell size limits, as well as machine adjacency constraints. They used LINGO, an off-the-shelf optimization software, to optimally solve their mathematical model. Ahkioon et al. [4] developed a mathematical model for adding the flexibility and reliability of the machines in the design of a dynamic cellular manufacturing system. They proposed the use of contingency process routings to create a continuous-flow system while machines are broken down. They solved the mathematical model with the use of CPLEX optimization software. Safaei and Tavakkoli-Moghaddam [17] developed a multi-period cellular manufacturing system that aims at minimizing costs including material handling costs such as inter/intra-cell costs, reconfiguration, and inventory carrying, as well as subcontracting of the part demands. They also investigated the potential economic advantages of outsourcing over the internal production in their mathematical model. Results obtained showed that the outsourcing fragments including inventory, backordering, and subcontracting has convulsive effects on the cellular reconfiguration due to the addition and removal of the machines in each and every time period and the large portion of the part demands can be satisfied with the use of less time periods in the production horizon. They solved the model with the use of LINGO optimization software. Solimanpur and Elmi [18] proposed a mixed-integer linear mathematical model for the use of scheduling production activities in a cellular manufacturing system. They solved the model using the tabu search algorithm. Koufteros et al. [19] investigated the interactions between product development strategies including platform products, concurrent engineering, and manufacturing practices such as cellular manufacturing and setup improvement practices. Results obtained demonstrated that manufacturing practices play a crucial role in realizing the value of product development practices. Aljuneidi and Bulgak [20] and Aljuneidi [21] designed an integrated hybrid cellular manufacturing-remanufacturing considering reconfiguration in their mathematical model. Throughout the sensitivity analyses, they showed that the quality of returned products has a direct effect on the total costs and the total number of the returned products that need to be acquired. They solved their model with the use of CPLEX. Aljuneidi [21] and Aljuneidi and Bulgak [22] developed a detailed mathematical model for the operational and tactical planning of a hybrid cellular manufacturing-remanufacturing enterprise. They considered various recovery operations such as remanufacturing and recycling with the assumption of disposing of the “low-quality” returned products. They solved the mathematical model using CPLEX optimization software. Saxena and Jain [23] developed a comprehensive mathematical model with many pragmatic elements such as process routings, outsourcing, work-load balancing, material handling, facility layout, and machine adjacencies for designing a dynamic cellular manufacturing system considering the reliability of machines. They solved the model proposed with the use of LINGO optimization software. Ghezavati [24] presented a stochastic mixed-integer model to design a cellular manufacturing system under the supply chain consideration where suppliers are required to operate exceptional products. The main objective of his mathematical model was to design a manufacturing system to work as a part of a closed-loop supply chain. He applied a hybrid metaheuristic method combining a genetic algorithm (GA) and simulated annealing (SA) to solve the mathematical model for the large-sized instances, while he solved the model with the use of LINGO software for smaller instances. Ghezavati [25] presented a mathematical model for designing cellular manufacturing integrated with the production planning problem where holding and backordering costs as well as part demands were uncertain. He solved the mathematical model with the use of LINGO software together with the branch and bound (B&B) algorithm and benchmark algorithm. Aalaei and Davoudpour [26] developed a mathematical model toward designing a cellular manufacturing system considering strategic decisions in a closed-loop supply chain with labor assignment. They considered different manufacturing features such as multiple facility locations, multi-market allocations with production planning issues. They considered three demand scenarios for part types such as optimistic, pessimistic, and normal. To solve the mathematical model, they developed a robust optimization approach model. Raoofpanah et al. [27] presented a mathematical model in designing a cellular manufacturing system to reduce the environmental hazards caused by the transportation system using robust optimization. They applied Benders-decomposition for solving the model to optimality. Feng et al. [28] developed a detailed cellular manufacturing system considering a dynamic cellular scheduling problem with flexible routes and machine sharing. Their model aims at minimizing the total workloads and make span. They solved the small instances of their model using CPLEX. For solving the large-sized instances of the model, they applied a three-layer chromosome genetic algorithm (TCGA) to get near-optimal solutions. Aljuneidi [21] and Aljuneidi and Bulgak [29] developed a mathematical model for designing sustainable manufacturing as a part of a closed-loop supply chain. The objective function of their model aims at minimizing the carbon emissions and travel distances between each facility. They solved the model with the use of CPLEX.

### Hybrid manufacturing-remanufacturing

In recent years, designing hybrid manufacturing-remanufacturing systems has become a topic of substantial interest due to economic opportunities, social incentives, and environmental legislation. In addition to economic, social and environmental incentives, remanufacturing may provide companies with the benefits of having a “green image.” In this section, a review of relevant papers on hybrid manufacturing-remanufacturing systems is presented. We have studied several aspects of hybrid manufacturing-remanufacturing such as objectives, problem-solving approaches, collection, disassembly, remanufacturing used products, incorporation of remanufacturing activities into new product manufacturing, and the nature of applied parameters including deterministic and stochastic. Van Der Laan et al. [9] investigated the production planning and inventory control of the hybrid manufacturing-remanufacturing systems using push and pull control strategies. They compared push and pull controlled systems with the traditional systems without remanufacturing option. The results obtained revealed that the total costs of the system tend to be lower in traditional systems without having a remanufacturing option. However, the total costs of the system may be lower in the push and/or pull controlled system using remanufacturing if system uncertainties are under control. Van Der Laan et al. [10] investigated the effect of lead-time duration and lead-time variability on the total expected costs of the hybrid manufacturing-remanufacturing systems using push and/or pull control strategies. A numerical study demonstrated that the variability in manufacturing lead-time has a more significant influence on total expected costs of the system. Also, a larger remanufacturing lead-time and larger variability in the manufacturing lead-time may result in a cost decrease. Parkinson and Thompson [30] presented the exact definitions of different processes in reverse logistics and remanufacturing including refurbishing, reconditioning, etc., because these processes were used with relatively the same meaning in publications. They also specified that remanufacturing operations can be implemented either by Original Equipment Manufacturer (OEM) or Third Part Remanufacturer (TRP), but it is common for having a collaboration between an OEM and a TPR. Inderfurth [31] investigated the effect of uncertainty in demand and quantity of returned products in a hybrid manufacturing-remanufacturing system. He showed for a single-period mathematical model with stochastic demand and stochastic returns considering the manufacturing and remanufacturing lead-times, how manufacturing and remanufacturing decisions can be coordinated to maximize the expected profit. Results obtained revealed that ‘order-up-to policy’ with two parameters and two parameters function is optimal given the proportional cost and revenues in a hybrid manufacturing-remanufacturing system. Demirel and Gökçen [32] developed a multi-phase and multi-product mixed-integer mathematical model for a remanufacturing system including both forward and reverse flows. Their model encompasses taking different decisions including optimal production quantities and transportation of manufactured and remanufactured products along with the optimal locations of disassembly, collection and distribution facilities. They considered three scenarios for the quality of the returned products to be low, medium, and high, and they investigated the effects of the quality of the returned products on the objective function and decision variables. Results obtained revealed that companies should consider more motivations for customers and subcontractors to take back the returned products. They solved the model with the use of CPLEX software. Li et al. [33] studied a single-period and multi-product production planning and inventory control strategies of a remanufacturing system considering the arrival timing, quality, and quantity of the returned products to be uncertain. They used a stochastic dynamic programming approach to formulate the mathematical model considering stochastic demands and stochastic quality of the returned products over a finite planning horizon. Their key decision variable was the optimal quantity of the returned products so as to minimize the total costs including remanufacturing cost, holding cost for the returned and remanufactured products, as well as the backlog cost. The policy of iteration method in dynamic programming was used to find the optimal solution. Mutha and Pokharel [34] developed a multi-echelon mixed-integer mathematical model for designing a reverse logistic network. In their network consumers provide their end-of-life products for retailers wherein retailers have the responsibility of collecting used products and sending them warehouses for consolidation. Next, used products are sent to reprocessing centers to be disassembled, cleaned, tested and sorted for reuse, remanufacturing, spare parts markets and recycling. They assumed that a portion of capacities in different facilities are assigned for remanufacturing activities. They solved the mathematical model using GAMS optimization software. Doh and Lee [35] designed a remanufacturing system intending to maximize the total profits. They considered different tactical planning issues in their model such as the number of acquired returned products, the number of returned products to be disassembled, as well as the number of returned products to be disposed; and also, the production planning problem of the proposed remanufacturing system in coordination with the supply chain. They solved the model with the use of CPLEX software and two heuristics such as LP relaxation approach and one to zero approach. Results obtained indicated the superiority of the two heuristic approaches over CPLEX software for solving medium to large instances. Wang et al. [5] investigated the design of a hybrid manufacturing-remanufacturing system for the perishable products when the quantities of both demands and returns are considered to be uncertain. Their mathematical model aimed at minimizing the total costs of the hybrid system. Results obtained showed that under the mixed strategy in coordination of manufacturing, remanufacturing, and disposal simultaneously, total costs of the enterprise will be less than considering manufacturing, remanufacturing, and disposal alternatively. The total costs of the system will also be reduced significantly if the amounts of manufactured products and the proportions of the remanufactured components to the returned products are set optimally. They analyzed and solved the model with the use of numerical simulation. Chen and Abrishami [8] developed a mathematical model for the design of a hybrid manufacturing-remanufacturing with the same and shared limited resources. They developed an exact method namely Lagrangean relaxation to optimally solve their mathematical model. Baki et al. [36] solved a lot-sizing problem in a remanufacturing system with the use of the Wagner–Whitin approach. Hasanov et al. [37] developed a mathematical model for designing a hybrid manufacturing-remanufacturing system where both of the demands for new and remanufactured components can be back-ordered. Results obtained demonstrated that an inventory policy can be set up only if there are several production and remanufacturing batches in an interval. This can happen only if the sum of the setup costs including the additional components is significantly high. Fang et al. [38] proposed a dynamic mathematical model for the production planning of a hybrid manufacturing-remanufacturing system where the demands considered to be uncertain. They solved the mathematical model with the use of Lagrangian-relaxation approach. Results obtained showed that the proposed solution algorithm generates solutions with a negligible gap to the optimal solutions in an acceptable computational time. Su and Xu [39] developed a mathematical model to analyze the buffer allocation problem considering uncertainty in the quality of the returned products in designing a remanufacturing system. The objective function of their problem was to minimize the total costs of the remanufacturing system when the total buffer capacity is set to be the constraint. They considered the grading policy for quality of the returned products to prioritize the high-quality returned products when service is required. They used the N-policy in which an equipment will not be idle at any time, so it will be used in the other tasks until the number of tasks is more than N. They solved the model with the use of decomposition-expansion algorithm and numerical simulation. Results demonstrated that grading the quality of returns together with the application of N-policy can reduce the total costs of the remanufacturing system significantly. Kim et al. [40] studied the effect of adding the disposal option of the returned products in a hybrid manufacturing-remanufacturing system. Results demonstrated that the value of having remanufacturing and disposal at the same time will be higher than having these options individually in the manufacturing system. Their results also showed that the value of disposing option is on average higher than remanufacturing option. Guo and Ya [41] developed a stochastic programming model to determine the optimal manufacturing and remanufacturing lots considering a minimum quality level of returns. They assumed that the quality of returns has an exponential distribution. Results obtained revealed that it is promising to use higher remanufacturing cost to remanufacture the lower quality of recycled products to reduce the average total cost.

From our review, we found that designing sustainable manufacturing systems has received increasing attention in recent years. One of the recommended manufacturing systems to achieve sustainability in manufacturing systems is the cellular manufacturing system [3]. Although there are research works in the literature review related to the design of sustainable manufacturing systems such as Aljuneidi and Bulgak [20–22, 29] in which they ignore the design elements of manufacturing systems including operation sequences of the part types, process routings of the part types including main alternative routings and contingency alternative process routings, as well as reliability of machines, the mathematical model in this paper encompasses all the missing aforementioned design elements of manufacturing systems to improve the sustainability pillars in designing sustainable manufacturing systems. In designing sustainable manufacturing systems, hybrid manufacturing-remanufacturing systems can also be applied because of their social, economic and environmental effects. Hence, we developed a hybrid cellular manufacturing-remanufacturing model considering alternative process routings and contingency process routings. The proposed mathematical model in this paper, to the best of our knowledge, is the first detailed mathematical model to enhance the flexibility and reliability of the hybrid cellular manufacturing-remanufacturing system considering reliability of machines in a closed-loop supply chain as a preliminary step toward the design of sustainable manufacturing systems.

## Mathematical model

### Problem description

**Fig. 1:**
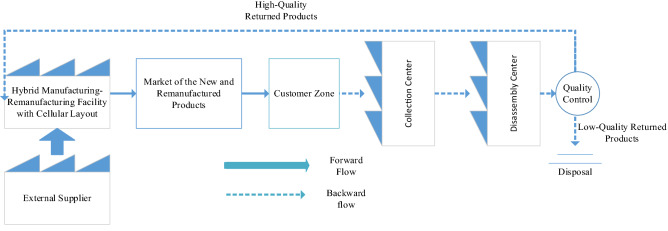
Material flow of the proposed cellular hybrid manufacturing-remanufacturing system

The design and optimization of a hybrid cellular manufacturing-remanufacturing system in a closed-loop supply chain configuration is presented in this section. Aljuneidi and Bulgak [20–22] previously proposed a mathematical model for the production planning of a hybrid cellular manufacturing-remanufacturing system. In this paper, the machine flexibility of the hybrid manufacturing-remanufacturing system is enhanced using alternative process routings. Alternative process routings can be formed when multi-functional machines and multiple copies of each machine type exist in the manufacturing system. Hence, each machine type can process different operations of a component. Likewise, each process of a component can be implemented on different machine types with different processing times. The mathematical model in this paper also considers one of the important manufacturing attributes namely, contingency process routings for all components together with the main process routings. Contingency process routings can be utilized when there are machine breakdowns or scheduled maintenance in the main process routings as components can immediately be re-routed when the main routings are unavailable. By taking contingency process routings into consideration, manufacturing systems can run in a continuous manner [4]. When contingency process routings are selected, the production of the other component types in the main routings is not be affected because the machines that are used in the main process routings in a time period are completely different entities from machines that are used in the contingency process routings. Hence, the contingency process routings can be used for the manufacturing of all the component types. In other words, the main process routings are the machines that are actively used to perform different operations of different part types. Contingency process routings as backup routings are through the machines that are simultaneously going to be set up for each of the part types. Contingency process routings are going to be activated when there is an interruption in one of the main process routings of a part type. In the event of having an interruption in the main process routings of a part, that part can be immediately re-routed to the contingency process routings. Hence, to enhance the reliability of the cellular manufacturing systems, contingency process routings can be used along with the main process routings. According to Fig. 1, in the forward supply chain, new components are manufactured with the use of raw materials. Remanufactured components are produced with the use of core components of the returned products as well. Accordingly, in the reverse chain, returned products are collected from the customer zones for inspection, classification, and testing in the collection center(s). Returned products are pulled apart in the disassembly center(s) to separate the remanufacturable and reusable components. In the quality control section of the disassembly center, components of the returned products are classified into two major categories which are “high-quality” and “low-quality” components. “High-quality” components are the ones with the high recovery rate (i.e. % 80 of the component is recoverable), while the “low-quality” components are the ones with the low recovery rate (i.e. less than % 10 of the component is recoverable) [42]. “High-quality” components are sent to the remanufacturing facilities in which the process of restoring to a “like-new” condition is performed. “Low-quality” components are going to be disposed. However, there are other recovery options such as reuse the returned products as they are, repair, refurbishment, and recycling that can be used for the returned products that have between 11 and 79% of recoverable components which is out of the scope of this paper. Remanufacturing usually consists of several stages including disassembly, cleaning, repairing, refurbishing and reassembling. Recognizing the proper manufacturing layout among flow-line production, job-shop production, or cellular manufacturing can highly improve the efficiency of the remanufacturing processes. To achieve sustainability in the manufacturing systems, cellular manufacturing layouts are highly recommended [3]. One of the most essential elements in designing cellular manufacturing systems is the consideration of machine reliability. In the literature review, the reliability of the machines is often considered to be 100%. In reality, machines fail during operations. Machine breakdowns are one of the key factors influencing the performance of the system at the operational level due to the causing probable postponements in the production planning of the manufacturing system. By taking machine reliability and breakdown effects of the machines to account at the operational level, solutions related to the selection of process routings with lower machine failures lead to the reduced overall cost of the cellular manufacturing systems [23, 43]. In this paper, it is assumed that the breakdown time for a machine of type *m* has an exponential distribution function with the failure rate equal to $$\lambda_{m}$$. Accordingly, reliability function *R* (*m*,$$t)$$ over the production time *t* can be written as:

$$R(m, t) = e^{{( - \lambda_{m} t)}}$$

Hence, number of the machine breakdowns can be calculated as:

$$N(m, t) = \frac{T}{{{\text{MTBF}}_{m} }},$$

**Fig. 2:**
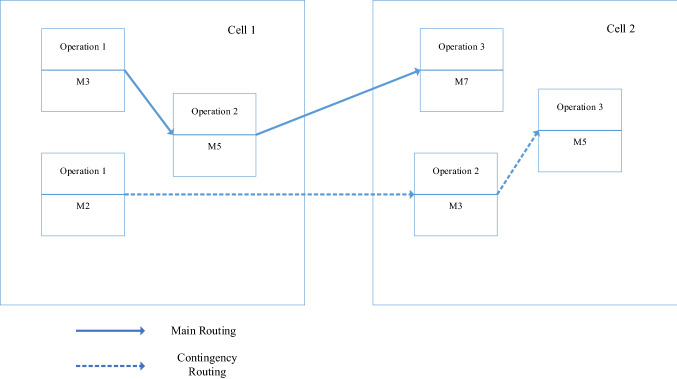
An example of main and contingency routings for a sample part in one period

*T* is the total production time and ${\text{MTBF}}_{m}$ is the mean time between failures of the machine type *m*. Breakdown cost of the machines can be calculated as:

$$\frac{T}{{{\text{MTBF}}_{m} }}*O_{m} ,$$

which $O_{m}$ represents the unit breakdown cost of the machine type *m*. Operational cost of the machines can be calculated as:

$$T*\left( {\frac{{{\text{MTBF}}_{m} + {\text{MTTR}}_{m} }}{{{\text{MTBF}}_{m} }}} \right)* \gamma_{m} ,$$

which $\gamma_{m}$ is the operational cost of the machine type *m*. The proposed model considers several manufacturing attributes such as multi-period production settings, reconfigurable layouts of the system, machine duplication, machine investment and machine capacity. There are several parameters pertaining to the reverse supply chain of the model including the acquisition of the returned products, disassembly of the returned products, remanufacturing of components having high qualities, as well as the disposition of the returned products that cannot be economically recovered. Figure 1 represents the material flow of the proposed hybrid cellular manufacturing-remanufacturing model. Figure 2 shows an example of the main and contingency process routings in the proposed model. When formulating the proposed mathematical model, several assumptions have been taken into consideration as follows:

- The number of cells is constant over the planning horizon and predefined.
- The demand for each component type is deterministic and known in advance in each time period.
- No backlogging is allowed.
- The demand for each component type in each time period can be fulfilled by internal production, and the inventories that can be carried over from the previous time period(s).
- Each machine type has a limited capacity expressed in hours during each time period.
- Reconfiguration involves the addition and removal of the machines to cells and relocation from one cell to another at the beginning of each time period.
- Lot-splitting and dynamic reconfiguration of the cells are considered.

The notations used for the model are presented below followed by the objective function and set of the constraints.

*Problem sets*:

*I*: Set of component types.
*M*: Set of machines.
*C*: Set of cells.
*T*: Set of time periods.
*J*: Set of returned products.
Ξ (*I*): Set of operation indices of part types.

*Parameters*

$$D_{it}$$
Demand of new component *i* in time period *t*.
$$D^{\prime}_{it}$$
Demand for remanufactured component *i* in time period *t*.
$$t_{\xi im}$$
Processing time of operation *ξ* of new component *i* on machine *m*.
$$t^{\prime}_{\xi im}$$
Processing time of operation *ξ* of remanufactured component *i* on machine *m*.
$$\pi_{m}$$
Time capacity of machine *m*.
$$\alpha_{c}$$
Lower size limit of the cells.
$$\beta_{c}$$
Upper size limit of the cells.
$$R_{m}$$
Installation cost of the machine *m*.
$$K_{m}$$
Removal cost of the machine *m*.
$$M^{\infty }$$
A large positive and integer number.
$$V_{it}$$
Holding/carrying cost of new component type *i* in time period *t*.
$$V^{\prime}_{it}$$
Holding/carrying cost of remanufactured component type *i* in time period *t*.
$$A_{m}$$
Machine availability in time period one before procuring any machines.
$$\psi_{m}$$
Machine maintenance and overhead costs.
$$\vartheta_{m}$$
Machine procurement cost.
$$\gamma_{m}$$
Operating cost of machine type *m*.
$$E_{i}$$
Production cost per new component type *i*.
$$E^{\prime}_{i}$$
Production cost per remanufactured component type *i*.
$$\omega_{jt}$$
Unit cost to acquire returned product *j* in time period *t*.
$$\phi_{jt}$$
Setup cost for disassembling returned product *j* in time period *t*.
$$\mu_{jt}$$
Unit cost to disassemble returned product *j* in time period *t*.
$$\lambda_{jt}$$
Unit inventory cost for returned product *j* in time period *t*.
$$\tau_{i}$$
Average recovering rate of part *i* from all returned products.
$$B_{ij}$$
Number of components *i* contained in product *j*.
$$F_{j}$$
Disposal cost of returned product *j*.
$$L_{\xi im}$$
If operation *ξ* of component *i* can be done on machine type *m*.
$${\text{MTBF}}_{m}$$
Average time between two consecutive failures of machine type *m*.
$${\text{MTTR}}_{m}$$
Average time to repair a failure of machine type *m*.
$$O_{m}$$
Breakdown cost for machine type *m*.

*Decision variables*

$$Y_{mct}^{ + }$$
Number of type *m* machines added in cell *c* at the beginning of time period *t*.
$$Y_{mct}^{ - }$$
Number of type *m* machines removed from cell *c* at the beginning of time period *t*.
$$\sigma_{mt}$$
Number of machines of type *m* procured at time *t*.
$$\hat{A}_{mt}$$
Quantity of machines type *m* available at time period *t* after accounting for machines that have been procured.
$$Q_{it}$$
Number of new components type *i* kept in inventory in time period *t* and carried over to period (*t* + 1).
$$Q^{\prime}_{it}$$
Number of remanufactured components *i* kept in inventory in time period *t* and carried over to period (*t* + 1).
$$d_{jt}$$
Number of returned products *j* to be disassembled in time period *t*.
$$r_{jt}$$
Number of returned products *j* to be acquired in time period *t*.
$$f_{jt}$$
Number of returned products *j* in inventory at the end of time period *t*.
$$\delta_{jt}$$
= 1, if returned product *j* will be disassembled in time period *t*, = 0, otherwise.
$$X_{\xi imct}$$
Number of new components of type *i* processed by operation *ξ* on machine *m* in cell *c* in time period *t* on the main routing.
$$Z_{\xi imct}$$
 = 1, if operation *ξ* of part type *i* is carried out on machine type *m* in cell *c* in period *t* on the main routing, = 0, otherwise.
$$X^{\prime}_{\xi imct}$$
Number of remanufactured components of type *i* processed by operation *ξ* on machine *m* in cell *c* in time period *t* on the main routing.
$$Z^{\prime}_{\xi imct}$$
= 1, if operation *ξ* of remanufactured component type *i* is carried out on machine type *m* in cell *c* in period *t* on the main routing, = 0, otherwise.
$$N_{mct}$$
Number of machines of type *m* presented in cell *c* at time period *t* for the main routing.
$$X^{\prime\prime}_{\xi imct}$$
Number of new components of type *i* processed by operation *ξ* on machine *m* in cell *c* in time period *t* on the contingency routing.
$$Z^{\prime\prime}_{\xi imct}$$
= 1, if operation *ξ* of component type *i* is carried out on machine type *m* in cell *c* in period *t* on the contingency routing.
$$X^{\prime\prime\prime}_{\xi imct}$$
Number of remanufactured components of type *i* processed by operation *ξ* on machine *m* in cell *c* in time period *t* on the contingency routing.
$$Z^{\prime\prime\prime}_{\xi imct}$$
= 1, if operation *ξ* of remanufactured component type *i* is carried out on machine type *m* in cell *c* in period *t* on the contingency routing; = 0, otherwise.
$$N^{\prime}_{mct}$$
Number of machines of type *m* presented in cell *c* at time period *t* for the contingency routing.

*Objective function*

$$\begin{gathered} \mathop \sum \limits_{t = 1}^{T} \mathop \sum \limits_{c = 1}^{C} \mathop \sum \limits_{m = 1}^{M} (N_{mct} + N_{mct}^{^{\prime}} )*\psi_{m} + \mathop \sum \limits_{t = 1}^{T} \mathop \sum \limits_{c = 1}^{C} \mathop \sum \limits_{m = 1}^{M} R_{m} * Y_{mct}^{ + } \hfill \\ + \mathop \sum \limits_{t = 1}^{T} \mathop \sum \limits_{c = 1}^{C} \mathop \sum \limits_{m = 1}^{M} K_{m} * Y_{mct}^{ - } + \mathop \sum \limits_{t = 1}^{T} \mathop \sum \limits_{i = 1}^{I} V_{i} * + Q_{it} \hfill \\ + \mathop \sum \limits_{t = 1}^{T} \mathop \sum \limits_{i = 1}^{I} V_{i}^{^{\prime}} *Q_{it}^{^{\prime}} + \mathop \sum \limits_{t = 1}^{T} \mathop \sum \limits_{c = 1}^{C} \mathop \sum \limits_{m = 1}^{M} \mathop \sum \limits_{i = 1}^{I} \mathop \sum \limits_{\xi = 1}^{\Xi } X_{\xi imct} *E_{i} \hfill \\ + \mathop \sum \limits_{t = 1}^{T} \mathop \sum \limits_{c = 1}^{C} \mathop \sum \limits_{m = 1}^{M} \mathop \sum \limits_{i = 1}^{I} \mathop \sum \limits_{\xi = 1}^{\Xi } X^{\prime}_{\xi imct} *E^{\prime}_{i} + \mathop \sum \limits_{t = 1}^{T} \mathop \sum \limits_{m = 1}^{M} \sigma_{mt} *\vartheta_{m} \hfill \\ + \mathop \sum \limits_{t = 1}^{T} \mathop \sum \limits_{c = 1}^{C} \mathop \sum \limits_{m = 1}^{M} \mathop \sum \limits_{i = 1}^{I} \mathop \sum \limits_{\xi = 1}^{\Xi - 1} X_{\xi imct} * t_{\xi im} *\left( {1 + \frac{{{\text{MTTR}}_{m} }}{{{\text{MTBF}}_{m} }}} \right)*\gamma_{m} \hfill \\ + \mathop \sum \limits_{t = 1}^{T} \mathop \sum \limits_{c = 1}^{C} \mathop \sum \limits_{m = 1}^{M} \mathop \sum \limits_{i = 1}^{I} \mathop \sum \limits_{\xi = 1}^{\Xi - 1} X^{\prime}_{\xi imct} * t^{\prime}_{\xi im} *\left( {1 + \frac{{{\text{MTTR}}_{m} }}{{{\text{MTBF}}_{m} }}} \right)*\gamma_{m} \hfill \\ \end{gathered}$$

$$\begin{gathered} + \mathop \sum \limits_{t = 1}^{T} \mathop \sum \limits_{c = 1}^{C} \mathop \sum \limits_{m = 1}^{M} \left( { \frac{{X_{\xi imct} * t_{\xi im} + X^{\prime}_{\xi imct} * t^{\prime}_{\xi im} }}{{{\text{MTBF}}_{m} }}} \right)*O_{m} \hfill \\ + \mathop \sum \limits_{t = 1}^{T} \mathop \sum \limits_{j = 1}^{J} \omega_{jt} *r_{jt} + \mathop \sum \limits_{t = 1}^{T} \mathop \sum \limits_{j = 1}^{J} \phi_{jt} *\delta_{jt} \hfill \\ + \mathop \sum \limits_{t = 1}^{T} \mathop \sum \limits_{j = 1}^{J} \mu_{jt} *d_{jt} + \mathop \sum \limits_{t = 1}^{T} \mathop \sum \limits_{j = 1}^{J} \lambda_{jt} * + f_{jt} \hfill \\ \mathop \sum \limits_{t = 1}^{T} \mathop \sum \limits_{i = 1}^{I} \mathop \sum \limits_{j = 1}^{J} \left( {1 - \tau_{i} } \right)* F_{j} * B_{ij} *d_{jt} \hfill \\ \end{gathered}$$

Subject to:

1.1 $$Q_{it - 1} + \mathop \sum \limits_{m = 1}^{M} \mathop \sum \limits_{c = 1}^{C} X_{\xi imct} - Q_{it} = D_{it} ;\quad \forall \left( {\xi ,i,t} \right)$$

1.2 $$Q^{\prime}_{it - 1} + \mathop \sum \limits_{m = 1}^{M} \mathop \sum \limits_{c = 1}^{C} X^{\prime}_{\xi imct} - Q^{\prime}_{it} = D^{\prime}_{it} ;\quad \forall \left( {\xi , \, i, \, t} \right)$$

1.3 $$Q_{it - 1} + \mathop \sum \limits_{m = 1}^{M} \mathop \sum \limits_{c = 1}^{C} X^{\prime\prime}_{\xi imct} - Q_{it} = D_{it} ;\quad \forall \left( {\xi , \, i, \, t} \right)$$

1.4 $$Q^{\prime}_{it - 1} + \mathop \sum \limits_{m = 1}^{M} \mathop \sum \limits_{c = 1}^{C} X^{\prime\prime\prime}_{\xi imct} - Q^{\prime}_{it} = D^{\prime}_{it} ;\quad \forall \left( {\xi , \, i, \, t} \right)$$

1.5 $$N_{mct} + N^{\prime}_{mct} = N_{mct - 1} + N^{\prime}_{mct - 1} + Y_{mct}^{ + } - Y_{mct}^{ - } ;\quad \forall \left( {m, \, c, \, t} \right)$$

1.6 $$\alpha_{c} \le \mathop \sum \limits_{m = 1}^{M} N_{mct} + N^{\prime}_{mct} \le \beta_{c} ;\quad \forall \left( {c, \, t} \right)$$

1.7 $$\mathop \sum \limits_{i = 1}^{I} \mathop \sum \limits_{\xi = 1}^{\Xi } X_{\xi imct} t_{\xi im} + X^{\prime}_{\xi imct} t^{\prime}_{\xi im} \le N_{mct} \pi_{m} ; \quad \forall \left( {m, \, c, \, t} \right)$$

1.8 $$\mathop \sum \limits_{i = 1}^{I} \mathop \sum \limits_{\xi = 1}^{\Xi } X^{\prime\prime}_{\xi imct} t_{\xi im} + X^{\prime\prime\prime}_{\xi imct} t^{\prime}_{\xi im} \le N^{\prime}_{mct} \pi_{m} ;\quad \forall \left( {m, \, c, \, t} \right)$$

1.9 $$\hat{A}_{{m\left( {t = 1} \right)}} = A_{{m\left( {t = 1} \right)}} + \sigma_{{m\left( {t = 1} \right)}} ;\quad \forall \left( m \right)$$

1.10 $$\hat{A}_{{m\left( {t + 1} \right)}} = \hat{A}_{mt} + \sigma_{{m\left( {t + 1} \right)}} ;\quad \forall \left( m \right)$$

1.11 $$\mathop \sum \limits_{c = 1}^{C} N_{mct} + N^{\prime}_{mct} = \hat{A}_{mt} ;\quad \forall \left( {m, \, t} \right)$$

1.12 $$X_{\xi imct} \le MZ_{\xi imct} ;\quad \forall \left( {\xi , \, i, \, m, \, c, \, t} \right)$$

1.13 $$X^{\prime}_{\xi imct} \le MZ^{\prime}_{\xi imct} ;\quad \forall \left( {\xi , \, i, \, m, \, c, \, t} \right)$$

1.14 $$X^{\prime\prime}_{\xi imct} \le MZ^{\prime\prime}_{\xi imct} ;\quad \forall \left( {\xi , \, i, \, m, \, c, \, t} \right)$$

1.15 $$X^{\prime\prime\prime}_{\xi imct} \le \, MZ^{\prime\prime\prime}_{\xi imct} ;\quad \forall \left( {\xi , \, i, \, m, \, c, \, t} \right)$$

1.16 $$f_{jt} + d_{jt} - f_{jt - 1} = r_{jt} ;\quad \forall \left( {j, \, t} \right)$$

1.17 $$d_{jt} \le M\delta_{jt} ;\quad \forall \left( {j, \, t} \right)$$

1.18 $$\mathop \sum \limits_{m = 1}^{M} \mathop \sum \limits_{c = 1}^{C} X^{\prime}_{\xi imct} \le \tau_{i} \mathop \sum \limits_{j = 1}^{J} B_{ij} d_{jt} ;\quad \forall \left( {j, \, t} \right)$$

1.19 $$\mathop \sum \limits_{m = 1}^{M} \mathop \sum \limits_{c = 1}^{C} X^{\prime\prime\prime}_{\xi imct} \le \tau_{i} \mathop \sum \limits_{j = 1}^{J} B_{ij} d_{jt} ;\quad \forall \left( {j, \, t} \right)$$

1.20 $$Z_{\xi imct} \le L_{\xi im} ;\quad \forall \left( {\xi , \, i, \, m, \, c, \, t} \right)$$

1.21 $$Z^{\prime}_{\xi imct} \le L_{\xi im} ;\quad \forall \left( {\xi , \, i, \, m, \, c, \, t} \right)$$

1.22 $$Z^{\prime\prime}_{\xi imct} \le L_{\xi im} ;\quad \forall \left( {\xi , \, i, \, m, \, c, \, t} \right)$$

1.23 $$Z^{\prime\prime\prime}_{\xi imct} \le L_{\xi im} ; \quad \forall \left( {\xi , \, i, \, m, \, c, \, t} \right)$$

1.24 $$\mathop \sum \limits_{m = 1}^{M} \mathop \sum \limits_{c = 1}^{C} X_{\xi + 1mct} = \mathop \sum \limits_{m = 1}^{M} \mathop \sum \limits_{c = 1}^{C} X_{\xi mct} ;\quad \forall \left( {\xi , \, i, \, t} \right)$$

1.25 $$\mathop \sum \limits_{m = 1}^{M} \mathop \sum \limits_{c = 1}^{C} X^{\prime}_{\xi + 1mct} = \mathop \sum \limits_{m = 1}^{M} \mathop \sum \limits_{c = 1}^{C} X^{\prime}_{\xi mct} ;\quad \forall \left( {\xi , \, i, \, t} \right)$$

1.26 $$\mathop \sum \limits_{m = 1}^{M} \mathop \sum \limits_{c = 1}^{C} X^{\prime\prime}_{\xi + 1mct} = \mathop \sum \limits_{m = 1}^{M} \mathop \sum \limits_{c = 1}^{C} X^{\prime\prime}_{\xi mct} ;\quad \forall \left( {\xi , \, i, \, t} \right)$$

1.27 $$\mathop \sum \limits_{m = 1}^{M} \mathop \sum \limits_{c = 1}^{C} X^{\prime\prime\prime}_{\xi + 1mct} = \mathop \sum \limits_{m = 1}^{M} \mathop \sum \limits_{c = 1}^{C} X^{\prime\prime\prime}_{\xi mct} ;\quad \forall \left( {\xi , \, i, \, t} \right)$$

1.30 $$N^{\prime}_{mct} ,N_{mct} ,Y_{mct}^{ + } ,Y_{mct}^{ - } \ge \, 0{\text{ and integer}};\quad \forall \left( {m, \, c, \, t} \right),$$

1.29 $$Q_{it} , Q^{\prime}_{it} \ge 0\quad \forall \left( {i, \, t} \right)$$

1.30 $$X_{\xi imct} ,X ^{^{\prime\prime}}_{\xi imct} , X^{^{\prime}}_{\xi imct} ,X^{\prime\prime\prime}_{\xi imct} \ge \, 0;\quad \forall \left( {\xi , \, i, \, m, \, c, \, t} \right)$$

1.31 $$\sigma_{mt} ,\hat{A}_{mt} \ge \, 0{\text{ and integer}};\quad \forall \left( {m, \, t} \right),$$

1.32 $$Z_{\xi imct} , Z^{\prime}_{zzimct} , Z^{\prime\prime}_{\xi imct} ,Z^{\prime\prime\prime}_{\xi imct} \in \left\{ {0, \, 1} \right\};\quad \forall (i, \, m, \, c, \, t).$$

*Model objective function*: The objective function of the model consists of several cost terms. The first term shows the maintenance and overhead costs of the machines involved in the main and contingency routings. The second term demonstrates the cost of the machine installations, while the third term represents the cost of the machine removals. The fourth term is pertinent to the inventory carrying cost of the components for manufacturing new products. The fifth term is pertinent to the inventory carrying cost of the components for remanufacturing returned products. The sixth term is relevant to the production cost of the new components. The seventh term is related to the production cost of the remanufactured components. The eighth term represents machines procurement cost. The ninth term demonstrates machine operating cost for producing new components. The tenth term shows machine operating cost for producing remanufactured components. The eleventh term shows the machine breakdown cost. The twelfth term represents acquiring cost of the returned products. The thirteenth term represents the setup cost for disassembling operations. The fourteenth term addresses the disassembling costs of the returned products. The fifteenth term shows the inventory holding costs for returned products, and the last term, term number sixteen, addresses the disposal cost of the returned products.

*Model constraints*: The objective function of the model is subjected to constraints as follows: constraint (1.1) demonstrates that the demands for a new component type *i* in each time period can be fulfilled by internal production, and/or the inventory that can be carried over from previous time period subtracting the inventory of the current time period in the main routing. Constraint (1.2) shows that the demands of a remanufactured component type *i* in each time period can be fulfilled by internal production, and/or the inventory that can be carried over from the previous time period subtracting the inventory of the current time period in the main routing. Constraint (1.3) demonstrates that the demands for a new component type *i* in each time period can be fulfilled by internal production, and/or the inventory that can be carried over from the previous time period subtracting the inventory of the current time period in the contingency routing. Constraint (1.4) shows that the demands for a remanufactured component type *i* in each time period can be fulfilled by internal production, and/or the inventory that can be carried over from the previous time period subtracting the inventory of the current time period in the contingency routing.

Constraint (1.5) demonstrates the reconfigurability of the manufacturing system where the number of machines of type m at the beginning of each time period is equal to the number of machines of type m in the previous time period considering installations and removals of the machines of type m in the cell c at the beginning of each time period. Constraint (1.5) ensures that machines can be chosen from the set of available machines in each time period to form the main or contingency routings. For example, a machine that was used in a period as part of a contingency routing is available for the next period to be used either in contingency or main process routings. Constraint (1.5) also ensures that within a period, machines allocated for the main routing will not be used for the contingency routing. The size of the cells is user-defined through constraint (1.6) where the number of machine assignments of each type should lie between the lower size and upper size of the cells. Constraint (1.7) ensures that the capacity of machines would not be exceeded for producing new and remanufactured components in the main routing. Constraint (1.8) ensures that the capacity of machines would not be exceeded for producing new and remanufactured components in the contingency routing. Constraint (1.9) is relevant to the availability of machines for time period 1 taking into consideration the machine procurements option. The total number of machines of each type available in the system is equal to the machine availability before machine procurements in addition to the number of machines acquired in the first time-period. Constraint (1.10) indicates that machine availabilities for the subsequent time periods excluding time period 1 can be recorded. The number of machine procurements in the current time period along with the number of machines that have been acquired in all the preceding time periods demonstrates total available machines in the system. Constraint (1.11) states that the total number of machines in each cell in both main routing and contingency routing should not exceed the total number of available machines. Constraint (1.12) indicates that the number of new components produced can be positive only if $Z_{\xi imct}$ = 1, that is, it has been decided that component *i* would be produced by operation $\xi$ on machine *m* in cell *c* in time period *t* in the main routing. Constraint (1.13) indicates that the number of remanufactured parts produced can be positive only if $ZR_{\xi imct}$ = 1, that is, it has been decided that remanufactured component *i* would be produced by operation $\xi$ on machine *m* in cell *c* in time period *t*. Constraint (1.14) indicates that the number of new components produced can be positive only if $ZP_{\xi imct}$ = 1, that is, it has been decided that component *i* would be produced by operation $\xi$ on machine *m* in cell *c* in time period *t* in the contingency routing. Constraint (1.15) indicates that the number of components produced can be positive only if $ZPR_{\xi imct}$ = 1, that is, it has been decided that remanufactured component *i* would be produced by operation $\xi$ on machine *m* in cell *c* in time period t in the contingency routing. Constraint (1.16) shows that the total number of returned products to be acquired can be calculated through the summation of the total number of returned products to be kept in inventory for the current time period as well as the total number of returned products to be disassembled for the current time period subtracting the amounts of inventory carried over from the previous time period. Constraint (1.17) indicates a logical constraint for disassembling activities. Constraint (1.18) takes to account the quality levels of the returned products. It represents the number of components acquired from returned products is dependent on their quality levels in the main routing. Constraint (1.19) takes to account the quality levels of the returned products. It represents the quantity of components acquired from returned products is dependent to their quality levels in the contingency routing. Constraints (1.20–1.23) ensures that each operation of a component either for the new components or remanufactured components is assigned to appropriate machines according to the part-machine incidence matrix in both main and contingency routings. Constraints (1.24–1.27) show the material flow conservation of the new and remanufactured components under production in both main and contingency process routings. This set of constraints ensures that the total quantity of parts processed in an operation is equal to the total number of components in the next/preceding operation. Constraints (1.24–1.27) also allow for the exploration of more allocations of the part routings. Constraint (1.28), Constraint (1.29), Constraint (1.30), Constraint (1.31) and Constraint (1.32) specify the logical binary and non-negativity integer requirements on the decision variables.

## Numerical example

A number of example problems are solved with the use of CPLEX, a commercially available software. For clarification purposes, one example problem is explained in detail to show the applications of the proposed model in designing a cellular hybrid manufacturing-remanufacturing system.

### Example 1

In solving Example 1, there are 2 components, 2 machines, 2 cells and 2 planning periods that have been considered. Also, in the reverse supply chain side of the model, there are 2 returned products that need to be collected from the customer zones.

#### Input data and problem size

The input data of Example 1 is given in Table 12 in Appendix. The input data are based on the work by Aljuneidi and Bulgak [20] and Zhou et al. [44]. Table 13 in Appendix shows the size of Example 1. Accordingly, there are 924 constraints as well as 704 integer variables.

#### Solution of Example 1

The main alternative process routings and contingency alternative process routings of the new components and remanufactured parts are brought in Tables 1, 2, 3 and 4. In Table 1, the main process routings of the new components are shown. According to Table 1, operation 2 of component type 1, for example, can be performed on machine type 1 in 2 different cells in time period 2. Hence, 339 components of type 1 are produced with the use of machine type 1 in cell 1 in time period 2 and 84 components of the same type are produced with the same machine type in cell 2 in the same time period. Table 2 demonstrates the contingency process routings for producing new parts. Accordingly, operation 4 of the component type 1 can be done on machine 1 and 2 in cell 1 in time period 1. Hence, 326 components of type 1 are produced with the use of machine type 1 in cell 1 in time period 1 and 51 components of the same type are produced with the use of machine type 2 in the same cell in the same time period. Noteworthy, the main routing for operation 4 of component type 1 in period 1 is the use of machine 1 in cell 2 with respect to Table 1. Therefore, 377 components of type 1 are produced with the use of machine 1 in cell 2 in time period 1 in the main routing. According to Table 2, operation 4 of component type 1 can be done on machine 1 and machine 2 through the use of cells 1 and 2 in case of unavailability of the main routing. According to Table 1, operation 4 of component type 1 is performed in the main routing on machine 1 in cell 1.

**Table 1:** Main routings for new parts

Part/operations	1	2	3	4	5
1	M2/C2/T1	M1/C2/T1	M1/C1/T1	M1/C2/T1	M2/C1/T1
	X1 = 377	X1 = 377	X1 = 377	X1 = 377	X1 = 124
					M2/C2/T1
					X1 = 253
	M2/C1/T2	M1/C1/T2	M1/C2/T2	M1/C1/T2	M2/C2/T2
	X1 = 423	X1 = 339	X1 = 423	X1 = 423	X1 = 375
		M1/C2/T2			M2/C1/T2
		X1 = 84			X1 = 48
2	M1/C1/T1	M2/C1/T1	M2/C2/T1	M1/C2/T1	
	X2 = 464	X2 = 464	X2 = 464	X2 = 443	M1/C1/T1
				M1/C1/T1	X2 = 464
				X2 = 21	
	M1/C1/T2	M2/C1/T2	M2/C1/T2	M1/C1/T2	M1/C1/T2
	X2 = 436	X2 = 436	X2 = 436	X2 = 436	X2 = 436

*M* Machine, *C* cell, *T* time period, *X* production rate for quantity components in the main routings

**Table 2:** Contingency routings for new parts

Part/operations	1	2	3	4	5
1	M2/C2/T1	M1/C1/T1	M2/C1/T1	M1/C2/T1	M2/C2/T1
	$X^{\prime}$1 = 377	$X^{\prime}$1 = 149	$X^{\prime}$1 = 377	$X^{\prime}$1 = 326	$X^{\prime}$1 = 377
		M1/C2/T1		2/C2/T1	
		$X^{\prime}$1 = 228		$X^{\prime}$1 = 51	
	M2/C2/T2	M1/C2/T2	M2/C2/T2	M1/C1/T2	M2/C1/T2
	$X^{\prime}$1 = 423	$X^{\prime}$1 = 423	$X^{\prime}$1 = 423	$X^{\prime}$1 = 298	$X^{\prime}$1 = 375
				M1/C2/T2	M2/C2/T2
				$X^{\prime}$1 = 32	$X^{\prime}$1 = 48
				M2/C2/T2	
				$X^{\prime}$1 = 98	
2	M1/C1/T1	M2/C2/T1	M2/C2/T1	M1/C1/T1	M2/C1/T1
	$X^{\prime}$2 = 464	$X^{\prime}$2 = 464	$X^{\prime}$2 = 434	$X^{\prime}$2 = 464	$X^{\prime}$2 = 464
			M2/C1/T1		
	M1/C2/T2	M2/C2/T2	$X^{\prime}$2 = 30	M1/C2/T2	M2/C2/T2
	$X^{\prime}$2 = 436	$X^{\prime}$2 = 436	M2/C2/T2	$X^{\prime}$2 = 436	$X^{\prime}$2 = 436
			$X^{\prime}$2 = 436		

*M* Machine, *C* cell, *T* time period, *X* production rate for quantity components in the main routings

**Table 3:** Main routings for remanufactured parts

Part/operations	1	2	3	4	5
1	M1/C1/T1	M1/C2/T1	M1/C2/T1	M1/C2/T1	M2/C2/T1
	$X^{\prime\prime}$ 1 = 107	$X^{\prime\prime}$ 1 = 107	$X^{\prime\prime}$ 1 = 107	$X^{\prime\prime}$ 1 = 107	$X^{\prime\prime}$ 1 = 107
	M1/C1/T2	M1/C1/T2	M1/C1/T2	M1/C2/T2	M2/C1/T2
	$X^{\prime\prime}$ 1 = 243	$X^{\prime\prime}$ 1 = 243	$X^{\prime\prime}$ 1 = 243	$X^{\prime\prime}$ 1 = 243	$X^{\prime\prime}$ 1 = 243
2	M2/C1/T1	M1/C2/T1	M1/C2/T1	M1/C2/T1	M1/C2/T1
	$X^{\prime\prime}$ 2 = 253	$X^{\prime\prime}$ 2 = 253	$X^{\prime\prime}$ 2 = 253	$X^{\prime\prime}$ 2 = 253	$X^{\prime\prime}$ 2 = 253
	M2/C1/T2	M1/C1/T2	M1/C1/T2	M1/C1/T2	M1/C1/T2
	$X^{\prime\prime}$ 2 = 297	$X^{\prime\prime}$ 2 = 297	$X^{\prime\prime}$ 2 = 297	$X^{\prime\prime}$ 2 = 297	$X^{\prime\prime}$ 2 = 297

*M* Machine, *C* cell, *T* time period, $X^{\prime\prime}$ production quantity for remanufactured components in the main routings

**Table 4:** Contingency routings for remanufactured parts

Part/operations	1	2	3	4	5
1	M2/C1/T1	M1/C2/T1	M1/C1/T1	M2/C1/T1	M2/C1/T1
	$X^{\prime\prime\prime}1$ = 107	$X^{\prime\prime\prime}1$ = 107	$X^{\prime\prime\prime}1$ = 107	$X^{\prime\prime\prime}1$ = 107	$X^{\prime\prime\prime}1$ = 107
	M2/C1/T2	M1/C1/T2	M1/C1/T2	M1/C2/T2	M2/C1/T2
	$X^{\prime\prime\prime}1$ = 243	$X^{\prime\prime\prime}1$ = 243	$X^{\prime\prime\prime}1$ = 243	$X^{\prime\prime\prime}1$ = 243	$X^{\prime\prime\prime}1$ = 243
2	M2/C1/T1	M2/C1/T1	M1/C1/T1	M1/C1/T1	M2/C1/T1
	$X^{\prime\prime\prime}2$ = 253	$X^{\prime\prime\prime}2$ = 253	$X^{\prime\prime\prime}2$ = 253	$X^{\prime\prime\prime}2$ = 253	$X^{\prime\prime\prime}2$ = 253
				M1/C2/T2	
	M2/C2/T2	M1/C2/T2	M2/C2/T2	$X^{\prime\prime\prime}2$ = 276	M1/C2/T2
	$X^{\prime\prime\prime}2$ = 297	$X^{\prime\prime\prime}2$ = 297	$X^{\prime\prime\prime}2$ = 297	M1/C1/T2	$X^{\prime\prime\prime}2$ = 297
				$X^{\prime\prime\prime}2$ = 21	

*M* Machine, *C* cell, *T* time period, $X^{\prime\prime\prime}$ production quantity for remanufactured components in the contingency routings

Table 3 shows the main process routings for producing remanufactured components. Table 4 also demonstrates the contingency process routings for remanufactured products. According to Table 3, operation 1 of component type 2, for example, is performed on machine type 2 in cell 1 in time period 2. Accordingly, 297 components of type 2 are produced with the use of machine type 2 in cell 1 in time period 2. However, in case of unavailability of the main routing due to the machine breakdowns for operation 1 of part type 2 in time period 2, re-routing will be implemented. Hence, operation 1 of component type 2 in time period 2 can be done on machine type 2 in cell 2 with respect to Table 4. According to Table 4, 297 components of type 2 are produced with the use of machine type 2 in cell 2 in time period 2. Table 3 demonstrates that operation 3 of part type 1, for instance, is performed in cell 2 with the use of machine type 1 with the quantity of 107 components in time period 1. Rerouting of the operation 3 of part type 1 in time period 1 can be done in case of having unavailability in the main routing. Hence, regarding Table 4, operation 3 of part type 1, with the same quantity, can be done on machine type 1 in cell 1.

Table 5 demonstrates the amount of inventory that needs to be kept in time period 1 for carrying it over to time period 2 for the new and remanufactured parts.

**Table 5:** Quantities of the inventory that needs to be kept

Part	Time period	Value for the new parts	Value for the remanufactured parts
1	1	177	7
1	2	0	0
2	1	164	3
2	2	0	0

According to Kusiak [45] and Leng et al. [6], resorting to alternative process routings decreases the total number of available machines in the system. Table 6 shows the number of machines in the system with the assumption of considering and eliminating alternative process routings. Accordingly, when machines are multi-functional and resorting to alternative process routings is available for the manufacturing systems, there are 64 machines in the system. However, with the assumption of eliminating alternative process routings, there are 74 machines in the system. Table 6 also shows when alternative process routing is eliminated from the mathematical model, the objective function value is increased from 3,333,489 to 3,636,369 which shows 302,880 in the objective function value. Accordingly, resorting to contingency process routings imposes a negligible increasing effect on the objective function value. According to Table 6, when contingency process routing is eliminated from the mathematical model 0.019% of the total costs are saved. Hence, objective function value is decreased from 3,333,489 to 3,332,851 by eliminating contingency process routings. This shows the efficiency of the proposed contingency routings in the mathematical model. Contingency process routings not only improve the efficiency and reliability of the system, but also impose an insignificant cost increases in the objective function value. Production managers dealing with designing reliable cellular manufacturing systems can resort to the advantages contained in the use of contingency process routings.

**Table 6:** Number of machines in the system

		Objective-function value	Objective function with the elimination of contingency routings	Percentage of increase
Number of machines with alternative routings	64	3,333,489	3,332,851	0.019%
Number of machines without alternative routings	74	3,636,369		

Tables 7 and 8 demonstrate the allocation of machines to cells in 2 time periods for the main and contingency process routings. According to Table 7, for example, there is a need for 8 machines of type 1 in cell 1 in the second time period. In Table 8, for instance, there is a need for 5 machines of type 1 in cell 2 in the second time period. It is worth noting the summation of the number of machines for a part type in a time period through the main and contingency process routings do not violate the upper limit cell size which is 20 machines. For example, the summation of the number of machines 1 in cell 1 and 2 in time period 1 in the main and contingency process routings is 19 which is < 20.

**Table 7:** Allocation and quantity of machine types for the main routing

Machines	Cells	Time periods	Value
1	1	1	5
1	1	2	8
1	2	1	5
1	2	2	2
2	1	1	2
2	1	2	4
2	2	1	4
2	2	2	2

**Table 8:** Allocation and quantity of machine types for the contingency routing

Machines	Cells	Time periods	Value
1	1	1	4
1	1	2	1
1	2	1	2
1	2	2	5
2	1	1	4
2	1	2	2
2	2	1	6
2	2	2	8

Table 9 shows the quantities of the returned products and the obtained components. Table 9 also shows the quantities of the returned products to be disassembled, quantities of the returned products that need to be kept in inventory as well as the values corresponding to the binary variable of the setup for disassembly operations. According to Table 9, there is a need to collect 22 returned products 1 from the customer zones in time period 1. However, in time period 2, there is a need for 24 returned product 1. Accordingly, there is no need for collecting returned product 2 in time period 1, but there is a need for the collection of 2 returned product 2 in time period 2. According to Table 9, the quantity of the returned products in the disassembly center is completely equivalent to the acquired quantity of the returned products for different time periods. The quantity of the returned products in the storage is 0 with respect to Table 9. Accordingly, the system is set up to perform the disassembly operations for the returned product 1 in time period 1 and time period 2 as well as for the returned product 2 in the second time period.

**Table 9:** Retuned products quantities

Returned product	Time period	Acquired quantity	Quantity in diss. ass. center	Quantity in inventory	Setup for diss. operations
1	1	22	22	0	1
1	2	24	24	0	1
2	1	0	0	0	0
2	2	2	2	0	1

## Computational experiments

To further illustrate the proposed model, we solved the mathematical model for seven other scenarios. According to Table 10, all the scenarios are solved with the use of IBM ILOG CPLEX Optimization Studio 12.7/OPL. For all the scenarios, the number of part types, the number of machines, the number of cells, and the number of returned products is reported. The number of constraints as well as the number of decision variables for each scenario is reported. The solution time is the major criterion for testing the solving ability of CPLEX. Seven scenarios are compared based on the computational times and the objective function value. For each scenario, the effect of the elimination of alternative process routings and contingency process routings on the objective function value is investigated in Table 11. The percentage of cost savings after the elimination of alternative routings can be calculated as 100* (objective function value without alternative routings—objective function with alternative process routings). Likewise, the cost savings related to the elimination of contingency process routings can be calculated as 100* (objective function value with contingency routings—objective function value without contingency process routings). Alternative process routings of the part types can be withheld by changing the part-machine incidence matrix. The mathematical model can be forced to select necessarily one machine at a time by assigning 0 to each operation of a part type that can be done on different machines on the part-machine incidence matrix. Contingency process routings can also be eliminated from the mathematical model by not considering the parameters, decision variables, objective function elements, and constraints related to the contingency (backup) process routings. According to Table 10, both solution times and objective function values are increased while increasing the scenario sizes. Scenario 1 can be solved within less than a minute. However, from scenario 2, the solution time is increased until the scenario 5 which takes 13 min to be solved. Based on our previous observations of the cellular manufacturing systems and with respect to the literature review, scenario 6 can be considered as a real-size instance of the mathematical model with 43,848 variables and 47,832 constraints. Scenario 6 can be solved in 47 min which shows a reasonable computational time. Scenario 7 is a large-scale instance of the mathematical model which cannot be solved in the polynomial-time by having 182,250 decision variables as well as 189,340 constraints. According to Table 11, by adding alternative process routings, 1.42% of the total costs can be saved on average. The maximum cost saving is relevant to scenario 6 with 2.94% and the minimum cost saving is pertinent to the scenario 1 with 0.88%. Hence, adding alternative process routings reduces the total costs of all the problem scenarios of the mathematical model developed in this paper regarding Table 11. According to Table 11, removing the contingency process routings has a very negligible effect on the total costs which shows the efficiency contained in designing contingency routings. Resorting to contingency process routings can increase the reliability and flexibility of the manufacturing systems against demand changes or machine breakdowns. According to Table 11, removing the contingency process routings can only decrease the total costs by 0.013%. Accordingly, the maximum cost saving is related to scenario 1 with 0.019% and the minimum cost saving is related to scenario 3 with 0.009%. Therefore, adding contingency process routings not only increases the reliability and routing flexibility of the mathematical model developed in this paper but also adds to the affordability of considering such routings with regard to a negligible increase in total costs of different problem scenarios of the mathematical model in Table 11.

**Table 10:** Different problem scenarios

Problem scenario	Number of component types	Number of time periods	Number of operations	Number of machine types	Number of products	Number of cells	Number of variables	Number of constraints	Solution time (s)	Objective function
1	2	2	5–9	2	2	4	1376	1612	28	3,333,494
2	2	3	5–9	3	2	2	2322	2700	89	4,160,333
3	4	3	5–9	3	3	3	7962	9312	194	17,249,057
4	4	3	5–9	3	3	5	13,218	14,598	363	17,282,646
5	4	6	5–9	3	3	3	15,924	18,624	767	33,809,026
6	5	6	5–9	5	2	4	43,848	47,832	2775	40,786,386
7	5	10	5–9	5	2	10	182,250	189,340	NA	NA

**Table 11:** Solution analyses for different problem scenarios

Problem scenario	Objective function without alternative routings	Percentage of cost saving by adding alternative process routings	Objective function without contingency routings	Percentage of cost saving with the elimination of contingency process routings
1	3,636,348	0.88	3,332,851	0.019
2	4,206,909	1.11	4,159,661	0.016
3	17,462,365	1.22	17,247,403	0.009
4	17,492,411	1.19	17,279,629	0.017
5	34,214,755	1.18	33,805,432	0.01
6	42,024,113	2.94	40,781,506	0.011
7	NA	NA	NA	NA
Average		1.42		0.013

Input data related to problem scenarios 1–2, 3–5, and 6–7 are brought in Appendix in Tables 14, 15, and 16, respectively. Table 14 in Appendix demonstrates input data related to problem scenarios 1 and 2 that have been generated randomly based on the research papers by Defersha and Chen [7] and Demirel and Gökçen [32]. Table 14 in Appendix shows input data related to problem scenarios 3–5 that have been generated randomly based on the research paper by Aljuneidi and Bulgak [22]. Table 16 in Appendix shows input data related to problem scenarios 6 and 7 that have been generated randomly based on the research paper by Chen and Abrishami [8].

## Conclusion and future research

In spite of the growing research interest in the design of the sustainable closed-loop supply chains, sustainability criteria in the design of manufacturing systems have absorbed less attention. In order to build a sustainable manufacturing enterprise incorporating the manufacturing system and its closed-loop supply chain, we presented a mixed-integer programming model for the design of a hybrid cellular manufacturing-remanufacturing model. This is, to the best of the authors’ knowledge, the first integrated model in the design of hybrid cellular manufacturing systems which considers alternative process routings and contingency process routings to enhance the flexibility and reliability of the manufacturing system. Several activities are considered in the closed-loop supply chain of the model including setup for disassembly of the returned products, disassembly of the returned products, remanufacturing of the returned products with the “high-quality” levels, and the disposal of the “low-quality” returned products. In the manufacturing system, the main activities are assigning machines to cells and assigning operations of each product to different machines in a cellular hybrid manufacturing-remanufacturing system. The objective function of the model is to minimize the total costs incorporating total costs related to the tactical planning of the closed-loop supply chain and costs related to the operational planning of the hybrid cellular manufacturing-remanufacturing system. Results obtained revealed that adding alternative process routings always improve the objective function value. On the other hand, resorting to contingency process routings increased the reliability and flexibility of the manufacturing system, proposed in this paper, against demand changes or machine breakdowns. Therefore, adding contingency process routings not only increases the reliability and flexibility of the mathematical model developed in this paper but also adds to the affordability of considering such routings with regard to a negligible increase in total costs. The future work in this research will be the development of appropriate solution techniques with the use of metaheuristics or exact methods such as Benders-decomposition or Lagrangian-relaxation. Considering stochastic parameters is another research direction that would be perused for future research. Also, considering the uncertainty in quality of returned products such as end-of-life and end-of-use products (cores) with the use of a random variable like Bernoulli or Exponential distribution would be perused for the future research.

## Appendix

See Appendix Tables

**Table 12:** Input data for Example 1

Parameter	Parameter setting/range (Units)
Demand for new part type *i* at time period *t*	200–600
Demand for remanufactured part type *i* at time period *t*	100–300
Processing time of operation *ξ* of new part type *i* on machine *m* (s)	4–8
Processing time of operation *ξ* of remanufactured part type *i* on machine *m* (s)	2–4
Production cost per new part type *i*	200–300
Production cost per remanufactured part type *i*	100–150
Inventory holding cost per new part type *i* per time period	8–10
Inventory holding cost per remanufactured part type *i* per time period	5
Quantity of machine type *m* available at time period *t*	6
Capacity of one unit of machine type *m* during one-time period	1500
Operating cost per unit time per machine type *m*	10
Maintenance and overhead costs per machine type *m*	20
Procurement cost per machine type *m*	500–700
Machine installation cost	10
Machine removal cost	8
Upper limit cell size	20
Lower limit cell size	3
Acquiring cost of the returned product *j* in time period *t*	1–3
Setup cost for disassembling returned product *j* in time period *t*	2–3
Inventory cost for returned product *j* in time period *t*	4–5
Disassembly cost of returned product *j* in time period *t*	2–3
Average recovering rate of returned product *j*	0.9
Number of parts *i* contained in product *j*	9–13
Disposal cost of the returned product *j*	60–70
Breakdown cost for machine type *m*	1200–1800
Average time between two consecutive failures of machine type *m* (min)	3–8
Average time between two consecutive repairs of machine type *m* (min)	51–90

^12^,

**Table 13:** Generic attributes of the Example 1

No. of parts	No. of machines	No. of cells	No. of retuned products	No. of operations	No. of binary variables	No. of integer variables	No. of constraints
2	2	2	2	5	324	704	924

^13^,

**Table 14:** Input data for Scenarios 1 and 2

Parameter	Parameter setting/range (units)
Demand for new part type *i* at time period *t*	4000–6000
Demand for remanufactured part type *i* at time period *t*	2500–3500
Processing time of operation *ξ* of new part type *i* on machine *m* (s)	40–100
Processing time of operation *ξ* of remanufactured part type *i* on machine *m* (s)	10–30
Production cost per new part type *i*	80–100
Production cost per remanufactured part type *i*	20–40
Inventory holding cost per new part type *i* per time period	5
Inventory holding cost per remanufactured part type *i* per time period	0.2–2.5
Quantity of machine type *m* available at time period *t*	6
Capacity of one unit of machine type *m* during one-time period	1800–2200
Operating cost per unit time per machine type *m*	5–15
Maintenance and overhead costs per machine type *m*	300–900
Procurement cost per machine type *m*	11,000–18,000
Machine installation cost	70–140
Machine removal cost	70–140
Upper limit cell size	25
Lower limit cell size	2
Acquiring cost of the returned product *j* in time period *t*	0.5–1
Setup cost for disassembling returned product *j* in time period *t*	0.5
Inventory cost for returned product *j* in time period *t*	0.2–0.5
Disassembly cost of returned product *j* in time period *t*	2–8
Average recovering rate of returned product *j*	0.1–0.2
Number of parts *i* contained in product *j*	3
Disposal cost of the returned product *j*	1–2
Breakdown cost for machine type *m*	1000–2000
Average time between two consecutive failures of machine type *m* (min)	3–5
Average time between two consecutive repairs of machine type *m* (min)	40–60

^14^,

**Table 15:** Input data for scenarios 3–5

Parameter	Parameter setting/range (units)
Demand for new part type *i* at time period *t*	200–600
Demand for remanufactured part type *i* at time period *t*	100–300
Processing time of operation *ξ* of new part type *i* on machine *m* (s	4–8
Processing time of operation *ξ* of remanufactured part type *i* on machine *m* (s)	2–4
Production cost per new part type *i*	200–300
Production cost per remanufactured part type *i*	100–150
Inventory holding cost per new part type *i* per time period	8–10
Inventory holding cost per remanufactured part type *i* per time period	5
Quantity of machine type *m* available at time period *t*	6
Capacity of one unit of machine type *m* during one-time period	100–500
Operating cost per unit time per machine type *m*	50
Maintenance and overhead costs per machine type *m*	40–70
Procurement cost per machine type *m*	300–400
Machine installation cost	40–80
Machine removal cost	40–80
Upper limit cell size	20
Lower limit cell size	3
Acquiring cost of the returned product *j* in time period *t*	30–80
Setup cost for disassembling returned product *j* in time period *t*	150–250
Inventory cost for returned product *j* in time period *t*	5–6
Disassembly cost of returned product *j* in time period *t*	50–80
Average recovering rate of returned product *j*	0–1
Number of parts *i* contained in product *j*	9–15
Disposal cost of the returned product *j*	200–400
Breakdown cost for machine type *m*	1200–1800
Average time between two consecutive failures of machine type *m* (min)	3–8
Average time between two consecutive repairs of machine type *m* (min	40–85

^15^ and

**Table 16:** Input data for scenarios 6 and 7

Parameter	Parameter setting/range (units)
Demand for new part type *i* at time period *t*	35–65
Demand for remanufactured part type *i* at time period *t*	5–15
Processing time of operation *ξ* of new part type *i* on machine *m* (s)	100–150
Processing time of operation *ξ* of remanufactured part type *i* on machine *m* (s)	65–80
Production cost per new part type *i*	75–105
Production cost per remanufactured part type *i*	15–50
Inventory holding cost per new part type *i* per time period	100
Inventory holding cost per remanufactured part type *i* per time period	50
Quantity of machine type *m* available at time period *t*	6
Capacity of one unit of machine type m during one-time period	100–500
Operating cost per unit time per machine type *m*	50
Maintenance and overhead costs per machine type *m*	40–70
Procurement cost per machine type *m*	400
Machine installation cost	80
Machine removal cost	40
Upper limit cell size	10
Lower limit cell size	3
Acquiring cost of the returned product *j* in time period *t*	15–35
Setup cost for disassembling returned product *j* in time period *t*	20–35
Inventory cost for returned product *j* in time period *t*	30–50
Disassembly cost of returned product *j* in time period *t*	10–35
Average recovering rate of returned product *j*	0.2–0.7
Number of parts *i* contained in product *j*	1–15
Disposal cost of the returned product *j*	100
Breakdown cost for machine type *m*	1200–1800
Average time between two consecutive failures of machine type *m* (min)	3–8
Average time between two consecutive repairs of machine type *m* (min)	51–90

^16^.
